# Effect of Treatment with Heated Scallop Shell Powder on the Inactivation of Naturally Existing Bacteria and *Listeria monocytogenes* Inoculated on Chicken Meat

**DOI:** 10.3390/foods13030370

**Published:** 2024-01-23

**Authors:** Kiuta Omura, Emi Kaibara, Sae Yamaguchi, Hana Aoyagi, Mari Nishio, Kazuhisa Tomita, Jun Sawai

**Affiliations:** 1 Department of Nutrition and Life Science, Faculty of Health and Medical Sciences, Kanagawa Institute of Technology, 1030 Shimo-Ogino, Atsugi 243-0292, Kanagawa, Japan; 2 Faculty of Applied Biosciences, Kanagawa Institute of Technology, 1030 Shimo-Ogino, Atsugi 243-0292, Kanagawa, Japan

**Keywords:** heated scallop shell powder, calcium oxide, disinfection, antimicrobial activity, antibacterial activity, *Listeria monocytogenes*, chicken meat

## Abstract

This study investigated the efficacy of heated scallop shell powder (HSSP) treatment in preserving chicken thigh meat. Chicken thigh meat was treated with HSSP slurry (1% and 5%) for 60 min, and the variation in aerobic bacteria and coliform populations was assessed during refrigerated storage (10 °C). There was a substantial increase in aerobic bacteria, reaching nearly 7 log_10_ colony forming unit (CFU)/g following 7 days of refrigeration, in the untreated chicken meat. Conversely, the aerobic bacterial population of the HSSP-treated chicken was <5 log_10_ CFU/g. Coliform growth in the untreated chicken reached over 5 log_10_ CFU/g following 7 days. In contrast, the coliform population of the HSSP-treated chicken did not reach 5 log_10_ CFU/g at 1% HSSP concentration; it was suppressed to <4 log_10_ CFU/g at 5% concentration. *Listeria monocytogenes*, which can grow at low temperatures, was inoculated into the chicken meat (5 log_10_ CFU/g) treated with alcohol, which was followed by HSSP. In the untreated chicken, *L. monocytogenes* increased to 9 log_10_ CFU/g even when refrigerated for 7 days. However, in the chicken treated with 5% HSSP, *L. monocytogenes* was suppressed to approximately 3 log_10_ CFU/g. These findings reveal that HSSP treatment is an effective method for disinfecting meat, inhibiting bacterial growth, and enhancing preservation.

## 1. Introduction

Currently, although some scallop shells are repurposed for use in food additives and paints, the majority of them are categorized as industrial waste. In areas where scallops are harvested, the heavy metals in internal organs and the odor emanating from discarded shells have become notable pollution issues [1,2,3]. Sawai et al. [4,5] demonstrated that heating scallop shells at 800 °C or higher results in the conversion of calcium carbonate (CaCO_3_), the primary component of the shell, into calcium oxide (CaO), which exhibits antimicrobial properties. Application of these heated shell powders to food products can extend their shelf life. Furthermore, using discarded seashells as a useful resource can mitigate pollution problems. Similarly, other seashells such as oyster shells [6,7,8,9], surf clam shells [10], mussels [11,12], and blood cockle shells [13] have been found to exhibit antimicrobial properties following heat treatment.

A scallop shell powder heated at 1000 °C had almost the same antibacterial activity as CaO [5]. Heated seashells, such as heated scallop shells, have been reported to be effective against bacteria [5,14,15], fungi [16,17], heat-resistant bacterial spores [18,19], viruses [20,21,22], and biofilms [23,24,25,26,27]. Recently, manufactured heated scallop shell powder (HSSP) nanoparticles have shown higher antimicrobial activity than microparticles [20,28,29]. Furthermore, paints containing HSSP are nontransparent because the HSSP is microparticles. By using HSSP nanoparticles, paints with antimicrobial activity and high transparency could be developed [21]. Recently, medical applications related to HSSP nanoparticles have also been investigated. Ishihara et al. [30] showed that treatment with HSSP nanoparticles (0.2 wt%) can disinfect wounds. Ointments containing nanoparticles (0.2 wt%) were also found to be effective [31]. Thus, their application in the medical field is expected. It should be emphasized that the application of heated seashell powders is spreading.

There are several reports on the application of heated shell powder in the food sector, including fresh vegetables [32,33,34,35], fruits [36], sausages [37], fish [38], and food packaging materials [39]. Specifically, these powders are as effective as or more effective than sodium hypochlorite (NaOCl) treatment in terms of disinfecting and preserving fresh vegetables. However, few reports regarding the effects of these powders on the treatment and preservation of meat [15,40] have been found. Cagri-Mehmetoglu [40] reported that HSSP treatment significantly reduced the growth of *Listeria monocytogenes* or *Salmonella enteritidis* inoculated on chicken wings. Ro et al. [15] demonstrated that storing HSSP-supplemented meat patties with beef at 10 °C completely inhibited the growth of three pathogenic *Escherichia coli* strains. Therefore, in this study, as an application of HSSP on meat, the antimicrobial effect of HSSP treatment and its preservation effect during refrigerated storage on bacteria originally present in chicken thighs and inoculated *L. monocytogenes* were investigated.

## 2. Materials and Methods

### 2.1. HSSP

Natural Japan Co., Ltd. (Abashiri, Hokkaido, Japan) prepared HSSP (particle size, 4 µm) via heat treatment at 1200 °C. After opening the package containing the powder, it was stored in a desiccator.

### 2.2. HSSP Treatment of Chicken Thighs

#### 2.2.1. Preparation of Samples and Inoculation with Pathogens

Chicken thigh meat cut into approximately 20 g pieces was purchased from a city supermarket. The cut chicken meat was used without any pretreatment (chicken meat samples) to investigate the naturally existing total aerobic bacteria and coliform counts. The chicken meat sample without HSSP treatment, shown in Section 2.2.2, was used as a control when examining chicken thighs for naturally existing bacteria.

The bacteria were inoculated using the following procedure: *L. monocytogenes* ATCC (American Type Culture Collection) 19114, the inoculum organism was stored in a 10% glycerol solution at −80 °C. Then, the bacterial cells were thawed and preincubated in a nutrient broth (Eiken Chemicals Co., Ltd., Tokyo, Japan) at 37 °C for 20 h, washed (3000 rpm, 10 min), and resuspended in sterile 0.85% saline at a concentration of 10^9^ colony forming unit (CFU)/mL. The cut chicken meat (approximately 500 g) was soaked in 500 mL of 70% ethanol for 15 min, transferred to a colander, and allowed to stand on a clean bench for 1 h. The colander was soaked in 500 mL of sterile water to remove the alcohol remaining in the chicken meat (15 min). Next, the colander containing chicken meat was soaked in sterile water (500 mL) and inoculated with 1 mL of the bacterial suspension of *L. monocytogenes* ATCC 1911 (approximately 10^9^ CFU/mL) for 15 min. The colander containing the chicken meat was drained for 20 min, and *L. monocytogenes* was allowed to settle. The *L. monocytogenes*-inoculated chicken meat was used for sampling in this study. The abovementioned processes were performed on a clean bench at room temperature (25 °C ± 2 °C). The *L. monocytogenes*-inoculated chicken meat without HSSP treatment, shown in Section 2.2.2, was used as a control when inoculated with *L. monocytogenes*.

#### 2.2.2. HSSP Treatment

The HSSP treatment was performed according to the protocol described by Yamanaka et al. [7]. The HSSP was added to a sterilized stainless-steel vessel containing sterilized water (2.8 L) at a concentration of 1 wt/*v*% or 5 wt/*v*% and agitated using a magnetic stirrer at 500 rpm. A disinfected colander containing chicken meat samples or *L. monocytogenes*-inoculated chicken meat samples (approximately 100 g) was immersed in the HSSP slurry for 60 min. Subsequently, the HSSP-treated chicken meat samples were drained for 1 h. The abovementioned procedure was performed at 25 °C ± 2 °C on a clean bench.

Approximately 20 g of the HSSP-treated or untreated chicken meat was sampled and homogenized with 100 mL of sterile physiological saline for 1 min using a stomacher (Pro Media, SH-IIM; Elmex Ltd., Tokyo, Japan). Then, a 1 mL aliquot of the solution in a stomacher filter bag (Elmex) was serially diluted with sterile 0.85% saline and incubated with Standard Methods Agar (Eiken Chemicals), X-GAL Agar (Nissui Pharmaceutical Co., Ltd., Tokyo, Japan), and PALCAM *Listeria*-Selective Agar (Merck KGaA, Darmstadt, Germany) to count the total aerobic bacteria, coliforms, and *Listeria*, respectively. After incubation at 37 °C for 48 h, the bacterial colonies were counted. This was set as day 0. 

The drained chicken meat was stored in a polyethylene bag (Ziploc^®^, Asahi Kasei Home Products Co., Ltd., Tokyo, Japan) at 10 °C to investigate the storage quality of the chicken meat after treatment. Then, the populations of aerobes, coliforms, and *Listeria* present in chicken meat after 3, 5, and 7 days of storage were estimated using the procedure described above.

### 2.3. Color Measurement

The color change was measured on days 0, 3, 5, and 7 of storage at 10 °C for the untreated and HSSP-treated chicken meat samples without alcohol treatment. The Hunter color values (L*, a*, and b*) of the chicken meat surface were measured using a colorimeter (CR-400, Konica Minolta, Inc., Tokyo, Japan) at three different regions on the chicken meat’s surface.

### 2.4. Statistical Analysis

All experiments were performed in triplicate (*n* = 3). Data are presented as mean ± standard error. Furthermore, data were subjected to a two-way analysis of variance with Tukey’s test using BellCurve for Excel^®^ version 2.0.3 (Social Survey Research Information Co., Ltd., Tokyo, Japan); *p* < 0.05 was considered statistically significant.

## 3. Results and Discussion

### 3.1. Naturally Existing Bacteria

Variations in the aerobic and coliform populations of the chicken meat following HSSP treatment are shown in Table 1 and Table 2, respectively. The population of aerobic bacteria in the untreated chicken meat increased by one order of magnitude to over 5 log_10_ CFU/g on day 3, even during refrigeration, and increased by approximately two orders of magnitude to reach 6.7 log_10_ CFU/g on day 7. Conversely, the HSSP treatment (1% and 5%) maintained levels 1–3 orders of magnitude lower than those of the untreated group, even after 7 days.

The population of coliforms in the untreated chicken exceeded 5 log_10_ CFU/g by day 7. On the contrary, in the HSSP treatment, the coliforms increased over time but remained < 5 log_10_ CFU/g at 1% and <4 log_10_ CFU/g at 5%, even after 7 days (Table 2).

Based on previous reports, the antimicrobial effects of CaO are caused by its alkalinity (pH ≥ 12) as a result of hydration. In addition to alkalinity, reactive oxygen species (ROS) released from CaO are considered another antimicrobial mechanism [41], and their formation has been detected in HSSP, including CaO as the main component [14,42]. ROS are highly oxidizing free radicals with significant reactivity to numerous biomolecules [43]. These ions can be lethal to bacterial cells, which is probably because of the damage they cause to bacterial membranes, DNA, and proteins.

Photographs of the chicken meat following the HSSP treatment are shown in Table 3. In the untreated case, almost no change was observed from day 0 to even after 7 days. In contrast, the surface of the chicken meat treated with 1% and 5% HSSP turned white. Comparing days 0 and 7 of HSSP treatment, no change in color was visually observed.

A detailed examination of color change in the chicken meat due to the HSSP treatment was conducted by measuring the meat color via the L*a*b* color system using a colorimeter (Table 4). L* values increased significantly with the HSSP treatment (*p* < 0.05), indicating a change in color to white, whereas a* values decreased significantly with the HSSP treatment (*p* < 0.05), which is consistent with a decrease in redness. On the contrary, b* values differed between the untreated and HSSP-treated samples, with some showing a significant difference, depending on the sample date. Although difficult to visually observe, color changes during storage showed a gradual but significant increase (*p* < 0.05) in L* and a* values for the untreated chicken meat. However, no significant color changes in the L*, a*, or b* values were observed during storage for either the 1% or 5% HSSP-treated chicken meat.

### 3.2. Inoculated Pathogenic Bacteria

*Listeria monocytogenes*, a foodborne pathogen, has been frequently reported in ready-to-eat products because of its ability to survive and grow under refrigerated conditions [44]. Many outbreaks have been recorded [45]; the lethality (fatality rate) of severe listeriosis ranges from 20% to 30% [46]. Therefore, chicken samples inoculated with *L. monocytogenes* were prepared and treated with 5% HSSP, which was particularly effective in inhibiting naturally existing bacteria (Section 3.1).

Table 5 shows the changes in aerobic bacteria and *Listeria* counts in the HSSP-treated chicken thighs during refrigerated storage. A slight difference in the populations of *Listeria* and aerobic bacteria was observed in the controls, indicating that the inoculated *L. monocytogenes* accounted for most of the bacteria present in the chicken thighs because of the alcohol treatment. *L. monocytogenes* in the untreated meat (control) increased from 5 log_10_ CFU/g to 9 log_10_ CFU/g after inoculation at 7 days of refrigerated storage. In contrast, the 5% HSSP treatment decreased the populations of *L. monocytogenes* in chicken meat by approximately two orders of magnitude (day 0) and maintained the *L. monocytogenes* population during the storage period (*p* < 0.05). The difference from the control on day 7 was approximately six orders of magnitude.

Cagri-Mehmetoglu [40] reported that chicken wings inoculated with *L. monocytogenes* and *S. enteritidis* at 8 log_10_ CFU/g and treated with HSSP showed a reduction of three to five orders of magnitude, respectively. Yamanaka et al. [7] prepared fried chicken using chicken thighs treated with heated oyster shell powder; sensory evaluation revealed that the fried chicken prepared using chicken thighs treated with heated oyster shell powder was softer and tastier than that prepared using untreated chicken thighs. Furthermore, Mine et al. [47] reported that adding heated oyster shell powder to minced meat strengthened the binding power and suppressed weight loss after heating. As mentioned previously, the sensory evaluation was satisfactory, and no serious problems with the HSSP-treated meat were anticipated at this stage.

## 4. Conclusions

In this study, HSSP treatment effectively inhibited naturally existing bacteria and the inoculated *L. monocytogenes* in chicken thigh meat during cold storage (~7 days), indicating that HSSP treatment is a valuable meat disinfection method. However, there is growing concern about the accumulation of used antimicrobials and antiseptics in rivers and other sources, the development of drug resistance in environmental microorganisms, and the spread of drug-resistant genes [48,49,50,51]. The heated shell powder, whose main component is CaO, exhibits antimicrobial activity, and it is used to control microorganisms in food and the environment. When released into the environment, the heated shell powder absorbs CO_2_ and returns to its original shell component, CaCO_3_, which has no antimicrobial activity. Then, it returns to the sea through rivers. It may also be used as a component of shellfish and may be caught and landed again. Shells can be regarded as a circulating antimicrobial agent, which is a material associated with the SDGs. Apart from calcium fortification, heated shell powder can help food producers and consumers produce and consume wholesome food with a good taste.

## Figures and Tables

**Table 1:** Variation in naturally existing total aerobic bacterial population (log_10_ CFU/g) of chicken meat after HSSP treatment and storage at 10 °C.

Treatment	Aerobic Bacteria Population (log_10_ CFU/g)			
	Day 0	Day 3	Day 5	Day 7
No treatment (Control)	4.6 ± 0.3 ^a,A^	5.6 ±1.4 ^a,AB^	6.5 ± 1.3 ^a,B^	6.7 ± 1.0 ^a,B^
HSSP 1%	3.9 ± 0.2 ^a,A^	3.5 ± 0.4 ^b,A^	6.1 ± 0.1 ^a,B^	5.6 ± 0.1 ^b,C^
HSSP 5%	3.8 ± 0.4 ^a,A^	4.2 ± 0.1 ^b,AB^	5.1 ±1.7 ^b,B^	3.8 ± 1.9 ^c,AC^

Abbreviation: HSSP, heated scallop shell powder. Means in the same column followed by different letters (^a–c^) are significantly different (*p* < 0.05). Means in the same row followed by different letters (^A–C^) are significantly different (*p* < 0.05).

**Table 2:** Variation in naturally existing total coliform population (log_10_ CFU/g) of chicken meat after HSSP treatment and storage at 10 °C.

Treatment	Coliform Population (log_10_ CFU/g)			
	Day 0	Day 3	Day 5	Day 7
No treatment (Control)	3.9 ± 0.8 ^a,A^	5.0 ± 1.7 ^a,B^	5.4 ± 1.6 ^a,B^	5.4 ± 1.1 ^a,B^
HSSP 1%	3.5 ± 0.4 ^ab,A^	3.5 ± 0.2 ^b,A^	4.3 ± 0.1 ^b,B^	4.8 ± 0.1 ^a,B^
HSSP 5%	2.9 ± 0.3 ^b,A^	3.2 ± 1.0 ^b,AB^	3.9 ± 0.4 ^b,B^	3.6 ± 1.1 ^b,B^

Abbreviation: HSSP, heated scallop shell powder. Means in the same column followed by different letters (^a,b^) are significantly different (*p* < 0.05). Means in the same row followed by different letters (^A,B^) are significantly different (*p* < 0.05).

**Table 3:** Changes in color values of chicken meat after HSSP treatment and storage at 10 °C.

Treatment	Storage Time			
	Day 0	Day 3	Day 5	Day 7
No treatment (Control)	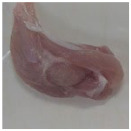	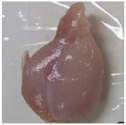	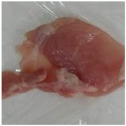	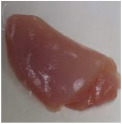
HSSP 1%	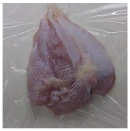	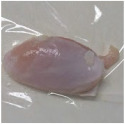	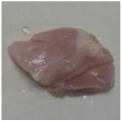	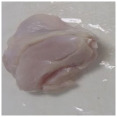
HSSP 5%	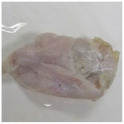	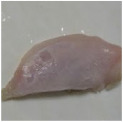	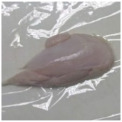	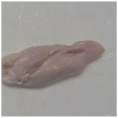

Abbreviation: HSSP, heated scallop shell powder.

**Table 4:** Changes in the Hunter color values of chicken meat after HSSP treatment and storage at 10 °C.

Hunter Color Values	Treatment	Storage Time			
		Day 0	Day 3	Day 5	Day 7
L*	No treatment (Control)	44.8 ± 5.2 ^a,A^	47.8 ± 4.3 ^a,AC^	51.6 ± 4.4 ^a,BC^	52.7 ± 5.2 ^a,BC^
	HSSP 1%	61.6 ± 9.4 ^b,A^	57.8 ± 5.9 ^b,A^	51.8 ± 1.6 ^a,B^	59.9 ± 5.6 ^b,A^
	HSSP 5%	61.6 ± 9.4 ^b,A^	57.8 ± 5.9 ^b,A^	51.8 ± 1.6 ^a,B^	59.9 ± 5.6 ^b,A^
a*	No treatment (Control)	2.9 ± 2.3 ^a,A^	4.8 ± 1.0 ^a,AB^	8.7 ± 0.3 ^a,D^	5.2 ± 1.9 ^a,CB^
	HSSP 1%	3.4 ± 1.4 ^a,A^	1.6 ± 0.8 ^b,A^	3.4 ± 2.5 ^b,A^	2.2 ± 2.9 ^b,A^
	HSSP 5%	0.9 ± 1.0 ^b,A^	0.9 ± 0.6 ^b,A^	1.7 ± 0.5 ^c,A^	2.6 ± 1.9 ^b,A^
b*	No treatment (Control)	5.1 ± 2.8 ^a,ABC^	2.8 ± 2.2 ^ab,B^	4.9 ± 4.3 ^a,ABC^	7.8 ± 2.6 ^a,C^
	HSSP 1%	4.7 ± 3.0 ^a,AC^	−0.2 ± 3.8 ^b,B^	2.6 ± 2.8 ^a,ABC^	5.5 ± 2.1 ^a,C^
	HSSP 5%	9.1 ± 5.1^b,A^	4.2 ± 2.7 ^ac,A^	8.5 ± 1.1 ^b,A^	7.9 ± 5.1 ^a,A^

Abbreviation: HSSP, heated scallop shell powder. Means in the same column followed by different letters (^a–c^) are significantly different (*p* < 0.05). L*, a*, and b* are statistically treated separately. Means in the same row followed by different letters (^A–D^) are significantly different (*p* < 0.05).

**Table 5:** Variation in the total aerobic bacterial population (log_10_ CFU/g) and *Listeria* population (log_10_ CFU/g) of chicken meat inoculated with *L. monocytogenes* after HSSP treatment and storage at 10 °C.

Bacteria	Treatment	Bacterial Population (log_10_ CFU/g)			
		Day 0	Day 3	Day 5	Day 7
Aerobic bacteria	No treatment (Control)	5.6 ± 0.2 ^a,A^	7.2 ± 0.8 ^a,B^	8.9 ± 0.4 ^a,C^	9.2 ± 0.2 ^a,C^
	HSSP 5%	3.6 ± 1.5 ^b,A^	4.2 ± 2.1 ^b,A^	4.4 ± 1.3 ^b,A^	3.5 ± 1.1 ^b,A^
*Listeria*	No treatment (Control)	5.5 ± 0.3 ^a,A^	7.0 ± 0.8 ^a,B^	7.1 ± 1.4 ^c,B^	9.0 ± 0.3 ^a,C^
	HSSP 5%	3.7 ± 1.4 ^b,A^	3.9 ± 1.6 ^b,A^	3.3 ± 0.9 ^b,A^	2.8 ± 0.2 ^b,A^

Abbreviation: HSSP, heated scallop shell powder. Means in the same column followed by different letters (^a–c^) are significantly different (*p* < 0.05). Means in the same row followed by different letters (^A–C^) are significantly different (*p* < 0.05).

## Data Availability

Data are contained within the article.

## Abbreviations

CFU	Colony forming unit
HSSP	heated scallop shell powder
*L. monocytogenes*	*Listeria monocytogenes*
ROS	reactive oxygen species
